# Effectiveness of the Antiparasitic Combination of Albendazole and Praziquantel As Compared With Albendazole Monotherapy in the Treatment of Neurocysticercosis in Children: A Systematic Review and Meta-Analysis

**DOI:** 10.7759/cureus.64617

**Published:** 2024-07-15

**Authors:** Dian Andriani Ratna Dewi, Lila Irawati Tjahjo Widuri, Arohid Allatib, Adristi A Athallah, Alessandro I Balga, Nabila Arkania, Farrasila Nadhira, Ni Made Wiliantari, Farida Ulfa

**Affiliations:** 1 Department of Dermatovenereology, Faculty of Military Medicine, The Republic of Indonesia Defense University, Bogor, IDN; 2 Department of Anesthesiology, Faculty of Military Medicine, The Republic of Indonesia Defense University, Bogor, IDN; 3 Department of Dermatovenereology, Faculty of Medicine, Public Health, and Nursing, Gadjah Mada University, Special Region of Yogyakarta, IDN; 4 Department of Dermatovenereology, Ratna Dewi Principal Clinic, Bekasi, IDN

**Keywords:** meta-analysis, systematic review, pediatric patients, monotherapy, combination therapy, praziquantel, albendazole, neurocysticercosis

## Abstract

Neurocysticercosis, caused by the tapeworm* Taenia solium*, is a neglected tropical illness that affects millions of people worldwide. The disease leads to seizures and epilepsy as the larvae invade the nervous system. Treatment with albendazole and praziquantel is common, but the comparative effectiveness of combination therapy versus monotherapy is unclear. This study evaluated the effectiveness of albendazole and praziquantel combination therapy versus albendazole monotherapy for lesion resolution in pediatric neurocysticercosis.

The study aimed to assess the effectiveness of the antiparasitic combination of albendazole and praziquantel as compared with albendazole monotherapy in the treatment of neurocysticercosis in children.

This study is based on a systematic review and meta-analysis following the Preferred Reporting Items for Systematic Reviews and Meta-Analyses (PRISMA) criteria. Randomized controlled trials on pediatric patients receiving the mentioned therapies were included. The risk-of-bias tool for randomized trials assessed the study quality once data extraction and analysis were completed.

This study included randomized research in neurocysticercosis pediatric patients diagnosed with neuroimaging outcomes, using albendazole and praziquantel combination therapy or albendazole monotherapy.

We searched articles between September 30 and December 1, 2023. All terms followed the Medical Subject Headings (MeSH) browser, and 13 articles were found. The data was quantitatively analyzed using RevMan 5.4.1 (The Nordic Cochrane Center, The Cochrane Collaboration, Copenhagen, Denmark). We applied the relative risk (RR) for the intervention and control groups before and after treatment, obtained from prior studies on lesion result resolution. The statistical method was Mantel-Haenszel. The model analysis we used was a fixed effect model (FEM) for heterogeneity (I2) < 50% and a random effect model (REM) for I2 ≥ 50%. The impact was measured using the risk difference (RD) by study and the overall 95% confidence interval (CI).

The meta-analysis indicated that combination therapy was more effective in achieving complete lesion resolution after both three months (pooled RD = 0.18, 95% CI = 0.03-0.33, p= 0.02, I2 =0%) and six months (pooled RD = 0.24, 95% CI = 0.09-0.40, p = 0.002, I2 =0%) of therapy. However, calcification outcomes were also more significant in the combination therapy group.

The study demonstrates that the albendazole and praziquantel combination therapy is superior in lesion resolution in pediatric neurocysticercosis. Clinical caution is advised to prevent calcification during treatment.

## Introduction and background

Pigs infected with *Taenia solium* tapeworm larvae are susceptible to neurocysticercosis (NCC). NCC is a neglected tropical illness that affects people worldwide, resulting in fatalities. As the worms infiltrate the human nervous system, the illness results in epilepsy. NCC frequently affects the cerebral cortex and cerebellum [1]. Rarely does NCC impact the pituitary gland. Cysts have the unique ability to group to create patterns resembling trees. Pituitary hormone insufficiency may result from a compression from a nearby cyst [2]. Eating or drinking water tainted with *Taenia solium* eggs or cysts can spread the disease [3].

**Figure 1:**
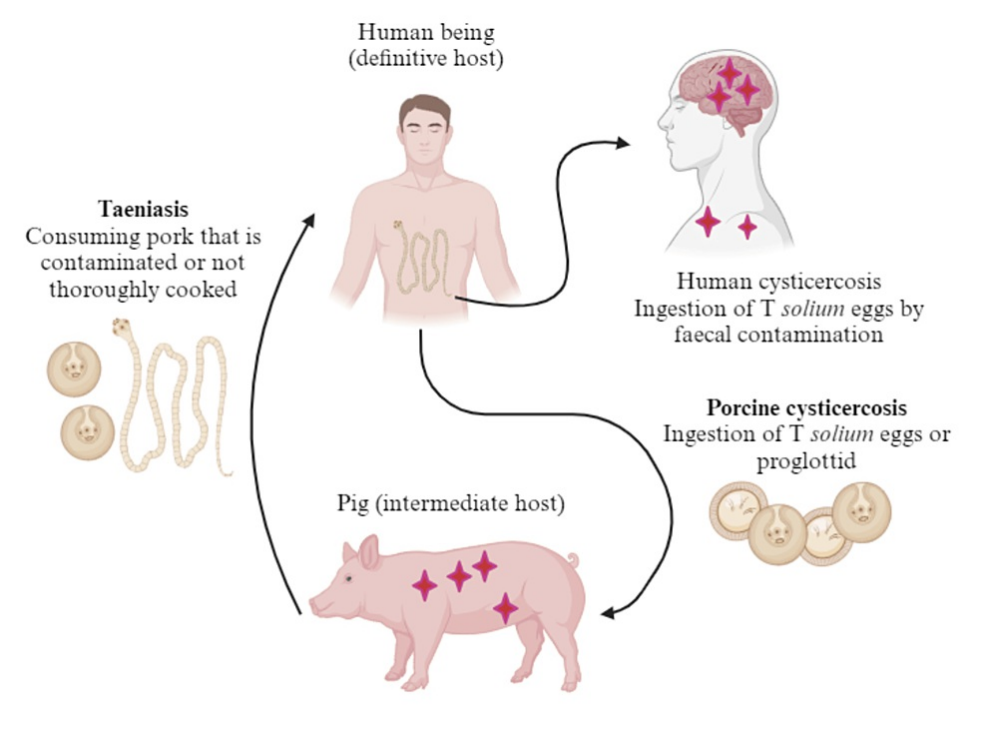
Pathogenesis of neurocysticercosis (NCC) The cysticerci enter the central nervous system via the lymphatic system or the circulatory system. They can cross the blood-brain barrier and spread throughout the spinal cord or brain. The subarachnoid space, the ventricular system, the brain parenchyma, and, in rare cases, the spinal cord are where cysticercosis development occurs most frequently in NCC. Image Credit: Author Arohid Allatib, using Biorender.com

When ingested, the cysts mature into adult worms in the human intestine. Adult worms will produce eggs, which are excreted in feces. If meat containing *Taenia solium* eggs is swallowed, it reaches the intestines and develops into oncospheres that have hooks [4]. The oncosphere larvae can pass through the intestinal wall with the help of these hooks. After that, the oncospheres can enter the portal blood vessels or lymph channels in the intestinal area, reaching the systemic circulation and finally entering the central nervous system (CNS) because they can pass through the capillary endothelium or choroid plexus epithelium. As a result, the person may suffer from NCC (Figure 1) [4,5].

The clinical aspects of NCC are determined by the location of the cysticercus in the CNS and the stage of infection due to changes in shape in the life cycle of the cysticercus. Cysticerci can infiltrate the blood-brain barrier and propagate throughout the spinal cord or brain. Cysticercosis most commonly affects the brain parenchyma, the ventricular system, the subarachnoid space, and, in rare cases, the spinal cord [6].

Complications of NCC include tissue damage due to immune cells releasing inflammatory mediators, disrupting the blood-brain barrier, and releasing toxic molecules. Immune cells such as microglia and macrophages are activated in response to the presence of parasites and components of the blood-brain barrier, such as endothelial and perithelial cells. They also produce anti-inflammatory cytokines that help regulate the body's immune response to infection. Alongside microglia and macrophages, various cytokines play significant roles in neurocysticercosis, including those with pro-inflammatory and anti-inflammatory properties, as well as cytokines involved in processes like angiogenesis, collagen deposition, and glial scar tissue formation. The immune system changes are observed in the nerve tissue adjacent to cysticerci [7]. The reaction of the CNS and the NCC can produce perilesional edema and swelling of the tissue around the cysticercus due to increased blood vessel permeability, disruption of the blood-brain barrier, and alterations in fluid control in the CNS [8]. It can even cause vasculitis [9]. 

NCC and its complications can result in neurological complications, including seizures, focal neurological deficits, cognitive impairment, hydrocephalus, vasculitis, spinal cord compression, increased intracranial pressure, and even life-threatening complications [10]. Granuloma formation and calcification in NCC can also be a host defense mechanism for containing and eliminating parasites. Granulomas usually consist of lymphocytes, macrophages, and multinucleated giant cells [11]. The clinical picture depends on the intricate linkages between the immune response, host-parasite interactions, and the location of the cysticercus inside the CNS [3]. Treatment depends on the disease's severity, cyst inflammation, degeneration degree, and potential complications. Praziquantel (PZQ) and/or albendazole (ABZ) are the drugs used for NCC. Both drugs can be given to children, but small doses of ABZ can be given to children over two years old. In clinical trials, ABZ therapy, together with dexamethasone, reduced inflammation and was shown to reduce the frequency of seizures for 30 months after treatment. In addition, ABZ may be better than PZQ at reducing *Taenia solium* worm larvae. However, in some patients, it may worsen symptoms of intracranial hypertension with cysticercosis encephalitis, a severe form of neurocysticercosis in which the brain parenchyma is harmed by the host immune response to a massive cysticerci infestation [12].

This study evaluated the effectiveness of ABZ and PZQ combination therapy compared with monotherapy using ABZ for lesion clearance in pediatric NCC, considering the previously mentioned background.

## Review

Methods

Research Methodology

The guidelines followed in this systematic review and meta-analysis study came from the Preferred Reporting Items for Systematic Reviews and Meta-Analyses (PRISMA) and the Cochrane Handbook for Systematic Reviews of Interventions, version 6.3, 2022.

Eligibility Criteria: Inclusion and Exclusion Criteria

Inclusion criteria included 1) randomized research, including randomized controlled trials and clinical trials, 2) pediatric patients diagnosed with NCC, 3) utilization of ABZ and PZQ combination therapy or ABZ monotherapy, 4) the evaluation of neuroimaging outcomes, and 5) adherence to the English language. On the other hand, exclusion criteria involved 1) the use of incompatible languages, 2) the unavailability of full-text access, and 3) the exclusion of pediatric patients according to the WHO terminology. The selection of titles and abstracts from the included papers was performed independently by three reviewers (AA, AIB, AAA), with any disagreements resolved through consultation with another author (DARD).

Standard of References 

The references were taken from a randomized controlled trial that examined the outcomes of ABZ monotherapy and combination therapy with PZQ in pediatric patients with NCC.

Search Strategy

**Figure 2:**
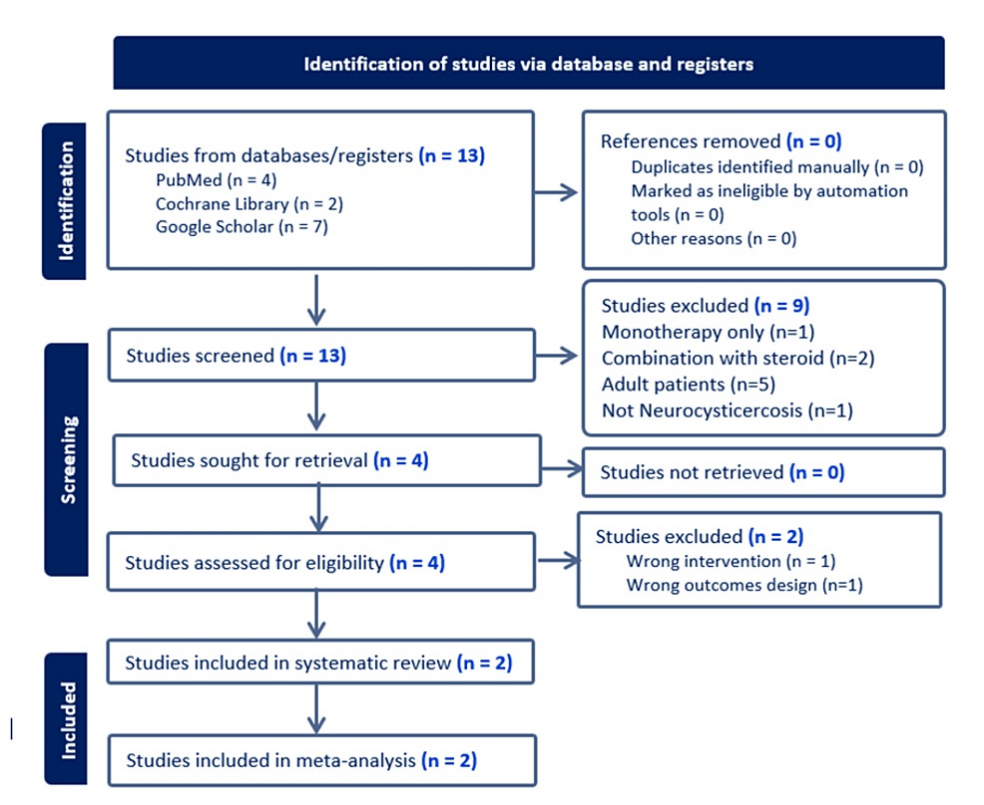
Preferred Reporting Items for Systematic Reviews and Meta-Analyses (PRISMA) flow diagram Image Credit: Author Arohid Allatib, using Biorender.com

As illustrated in the first attachment, the literature search encompassed databases such as PubMed and Cochrane. The searches were executed between September 30 and December 1, 2023. All terms followed the Medical Subject Headings (MeSH) browser. Keywords were added to the search area using Boolean operators. Specific terms were used according to the criteria of Boolean operator keywords, for example, ((((Neurocysticercosis [Title/Abstract]) AND (children [Title/Abstract]))) Neurocysticercosis (MeSH Terms) AND ((Praziquantel) AND (Albendazole)) AND (combination)) as seen in Figure 2 and Appendix 1.

Data Extraction and Analysis

A summary of the studies examined in this study is available in Table 1. Data gathered from the selected studies included the author, year, country, sample size, adverse event, and lesion resolution. The meta-analysis was carried out with RevMan version 5.4.1 (The Nordic Cochrane Center, The Cochrane Collaboration, Copenhagen, Denmark).

**Table 1:** Description of the included studies Credit: Author Dian Andriani Ratna Dewi

No	Author, year	Title (country)	Study design	Number, (combination/albendazole only)	Population characteristic	Intervention/comparison	Outcome
1.	Singh et al., 2022 [13]	Efficacy of combination therapy of albendazole and praziquantel vs albendazole monotherapy in children with persistent neurocysticercosis: a randomized controlled trial	Randomized controlled trial	40 (21/19)	Children five to 14 years old diagnosed with neurocysticercosis	Three groups of patients: (1) a group receiving albendazole monotherapy (for 30 days), (2) a combination therapy group (albendazole for 30 days + praziquantel for 15 days + placebo for the remaining 15 days), and (3) a placebo group (receiving 30 days of inert starch/lactose placebo). Albendazole 15mg/kg/day orally in two divided doses, praziquantel at 50 mg/kg/day orally in two divided doses, and placebo at 50 mg/kg/day orally in two divided doses.	In the initial month, one patient from group 1 and one from group 3 experienced a new episode. In six months, 62% in group 2 exhibited complete resolution and 26.3% in group 1 (p-value = 0.02). The percentage reduction in mean lesion area at six months was the highest in group 2 compared to the other groups (p-value = 0.006). The incidence of calcification remained consistent in the three groups (10%).
2.	Kaur et al., 2009 [14]	Combination therapy with albendazole and praziquantel versus albendazole alone in children with seizures and single lesion neurocysticercosis: a randomized, placebo-controlled double-blind trial (India)	Randomized controlled trial	103 (53/50)	Children one to 13 years old presented with seizures (focal or generalized) and the last seizure event occurred less than three months ago	Each child was given albendazole (15 mg/kg body weight per day, divided into three doses) for seven days and oral prednisolone ((2 mg/kg/d) for the first five days. Thereafter, all children were given different interventions, where the children were divided into two groups: group A and group B. Group A was treated with praziquantel, and group B was treated with placebo (75 mg/kg/day).	In total, 60% and 72% of infants at three and six months, respectively, in group 1 showed complete resolution; in group 2, 42% and 52%, respectively. However, nonresolution and calcification were more prevalent in group 2 than in group 1 at three months (Group 2: 28% and 14%, respectively; Group 1: 12% and 8%, respectively) and six months (Group 2: 16% and 22%, respectively; Group 1: 6% and 9%, respectively). Seizure management and adverse effects were comparable across both groups.

Risk of Bias in Individual Studies (Qualitative Synthesis)

**Figure 3:**
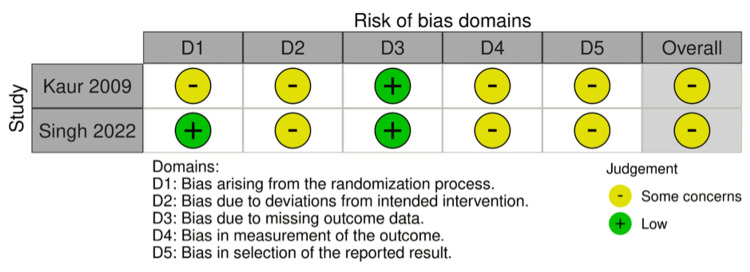
Randomised controlled trial risk of bias References: [13,14] Image Credit: Alessandro I. Balga, using Biorender.com

The risk-of-bias tool for randomized trials version 2 (RoB2), which is composed of five domains and 28 signaling questions concerning the randomization technique, intervention, outcome data, and reported results, was used to assess the quality of randomized controlled trials (Figure 3).

Quantitative Data Synthesis (Meta-Analysis)

The outcome variable of this quantitative data analysis evaluated the effectiveness of the antiparasitic combination of ABZ and PZQ compared with ABZ monotherapy in the treatment of NCC in children. The data was quantitatively analyzed using RevMan 5.4.1 (The Nordic Cochrane Center, The Cochrane Collaboration, Copenhagen, Denmark). We applied the relative risk (RR) for the intervention and control groups before and after treatment, which was obtained from prior studies on lesion result resolution. The data were dichotomous, and the statistical method was Mantel-Haenszel.

The model analysis we used was a fixed effect model (FEM) for heterogeneity (I2) < 50% and a random effect model (REM) for I2 ≥ 50%. Risk difference (RD) of lesion resolution from baseline using combination therapy and monotherapy for NCC patients was assessed as the major outcome in the statistical analysis, as shown by a substantial influence on lesion resolution. The impact was measured using the risk difference by study and the overall 95% confidence interval (CI).

Result and discussion

We included two studies, comprising 143 patients in total. The overall sample size was separated into two groups: 74 intervention patients and 69 control patients. Both studies were conducted in India. The extraction table summarizes each included study in Appendix 2.

ABZ's Mechanism of Action in Treating NCC

ABZ and PZQ are frequently used in the therapy of NCC. Both medications, when employed for NCC treatment, operate differently. ABZ is a benzimidazole-family anthelmintic drug that provides a broad range of uses against hydatid cysts and cysticercosis [15]. ABZ was first utilized for its antiparasitic properties in 1973 [16]. It is widely used in clinical chemotherapy to treat a range of human cystic echinococcosis and hydatid illnesses. In addition to cystic or hydatid disease, ABZ is the first-line treatment for microsporidiosis in HIV, enterobiasis, ascariasis, hookworm infections, and NCC [17].

**Figure 4:**
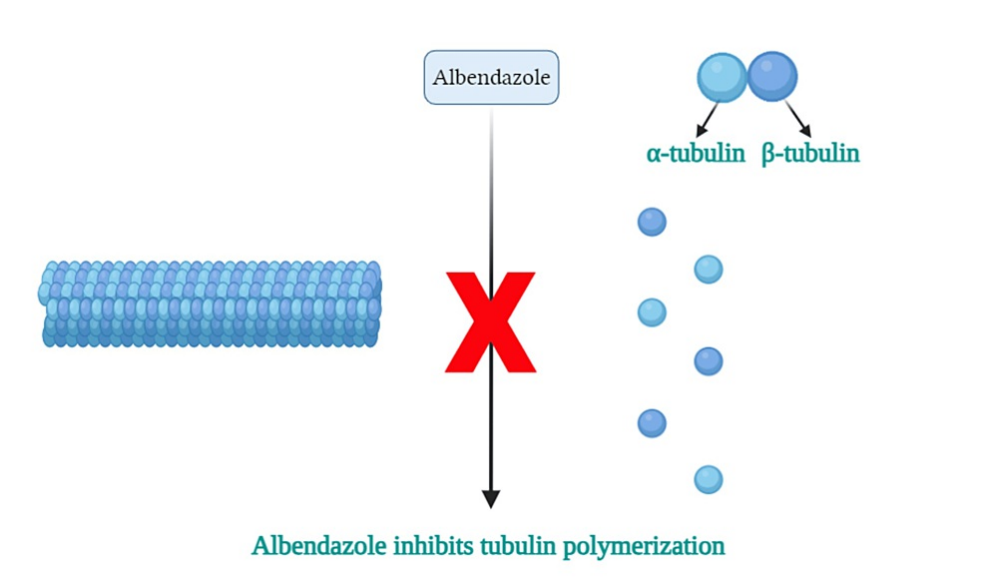
Albendazole's mechanism of action Image Credit: Author Nabila Arkania, using Biorender.com

The mechanism of action of ABZ is represented in Figure 4. ABZ inhibits tubulin polymerization. Tubulin-binding agents (TBAs) can inhibit cell motility by affecting microtubules; this affects the cytoskeletal organization and leads to vacuolization in the tegumental layer [18]. Microtubules are essential for the parasite's ability to invade host cells [19]. TBAs can bind to parasite tubulin and damage parasite microtubules [20]. This binding causes mitotic arrest during the metaphase/anaphase transition, which leads to cell death by apoptosis. ABZ is a tubulin polymerization inhibitor that specifically binds to β-tubulin through the colchicine site, blocking microtubule elongation and depolymerizing the subunits [21].

PZQ's Mechanism of Action

PZQ is a type of chemical substance generated from tetrahydrogenated isoquinoline, also called tetrahydroisoquinoline. PZQ is a class of organic compounds derived from tetrahydrogenated isoquinoline, also known as tetrahydroisoquinoline. The WHO recommends a single dosage of 40 mg/kg PZQ for the treatment of all forms of schistosomiasis; however, in some cases, a higher dose of 60 mg/kg PZQ has also been used [22,23]. 

**Figure 5:**
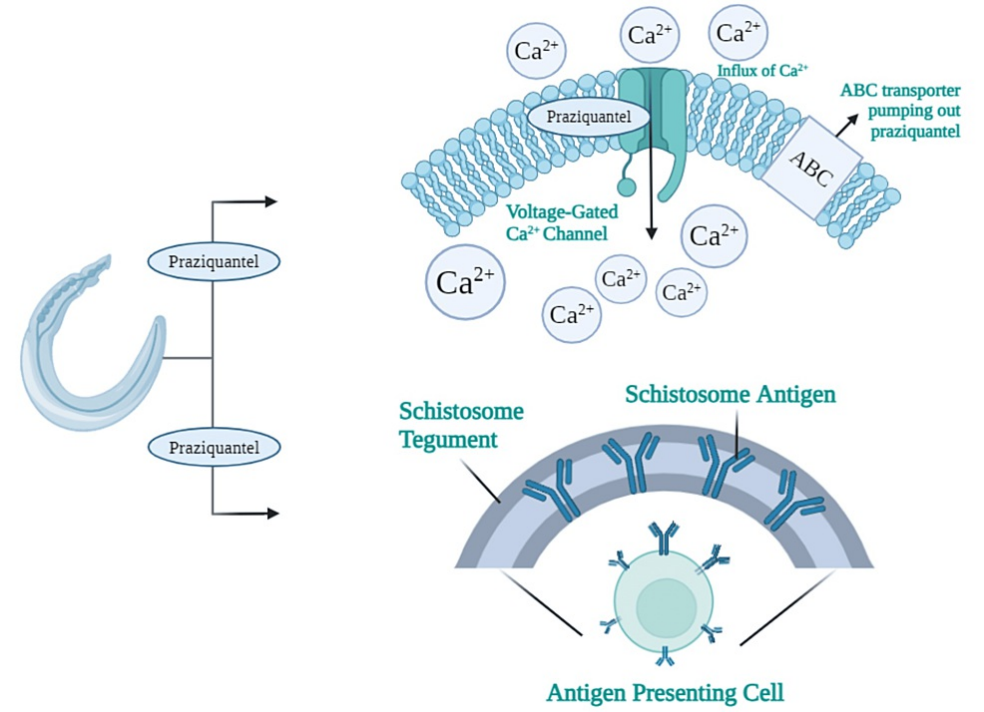
Praziquantel's mechanism of action Due to the absence of two conserved serine residues in the β subunits of voltage-gated Ca2+ channels, praziquantel was thought to target these subunits in Schistosoma mansoni and Schistosoma japonicum specifically. Ca2+: calcium Image Credit: Author Farrasila Nadhira, using Biorender.com

Muscle spastic paralysis occurs in schistosomes exposed to PZQ [24]. This contraction may be caused by a rapid influx of Ca2+ into the Schistosoma, the schistosomal calcium channel being a molecular target for PZQ. Schistosomes' β subunit differs from nine other β subunits, which may inhibit the flow of Ca2+. PZQ allows for the opening of additional channels, which disrupts Ca2+ homeostasis (Figure 5) [25]. 

PZQ causes morphological alterations in tegument worms, first characterized by tegument vacuolization and surface blebbing [26, 27]. This morphological alteration enhances the schistosome antigen's presentation on the parasite surface [28]. The impact of PZQ on exposed worm antigens could be due to its lipophilicity, which facilitates interaction with the hydrophobic core of the tegument. Furthermore, antigen exposure has been identified and confirmed to be linked to the host immune response, which is required for full PZQ function [27,29].

Matthaiou et al.'s meta-analysis's findings show that ABZ is superior to PZQ in managing seizures in afflicted individuals, causing cysts to vanish completely, and ultimately curing NCC patients [30].

PZQ concentrations are found to fall when steroids are taken, according to pharmacokinetic studies. One-day regimens with three doses of PZQ 25 mg/kg given at two-hour intervals were used in a Mexican trial [31]. Steroid delivery was postponed until peak PZQ concentrations could be maintained. Individuals with a single lesion responded better to this one-day treatment plan than individuals with several parasite cysts [32]. When ABZ was first used to treat NCC in 1987 [33], for a month, it was given every day at a dose of 15 mg/kg.

Further research has demonstrated that the effectiveness of treatments administered for 15 days or longer than seven days is comparable [33]. Additionally, ABZ exhibits faster lesion resolution and a better reduced risk of delayed seizure recurrence. Some literature also compares the efficacy between ABZ monotherapy and the combination therapy of PZQ and ABZ [34].

Combination therapy causes more significant and irreversible damage to the parasite tissue than administering drugs alone. This combination demonstrates higher efficacy within a shorter duration than each drug administered separately. This combination's effectiveness could be related to the additive interaction and synergistic effects of ABZ and PZQ. Furthermore, administering the combination does not affect the number or severity of the side effects. Therefore, combination therapy is superior to monotherapy [35].

We separated the meta-analysis in this review into smaller groups to produce more specific results based on the information from the included publications. Our meta-analysis results were classified by the duration of outcome measures and the feature of lesion resolutions.

**Figure 6:**
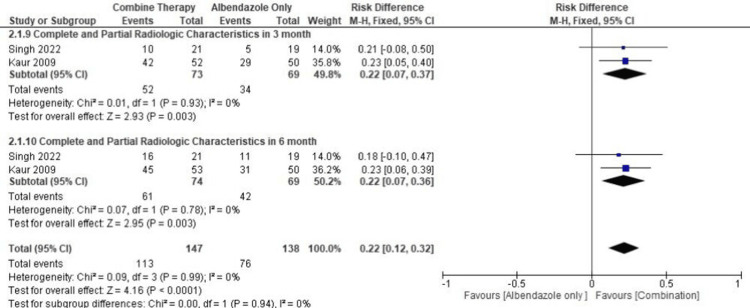
Meta-analysis for complete and partial radiological outcomes References: [13,14] Image Credit: Author Ni Made Wiliantari

In the first subgroup, we looked at the effectiveness of ABZ and PZQ combination therapy versus ABZ monotherapy in producing complete and partial radiological features after three months of therapy, in the two included articles, as seen in Appendix 2 [13,14]. Significant meta-analysis findings were found in a combination group after three months of therapy (pooled RD = 0.22, 95% CI = 0.07-0.37, p = 0.003, I2 =0%) (Figure 6).

In the second subgroup, we looked at the effectiveness of ABZ monotherapy compared with ABZ and PZQ combination therapy in producing complete and partial radiological features after six months of therapy. Significant meta-analysis results were found in a combination group after six months of therapy (pooled RD = 0.22, 95% CI = 0.07-0.36, p = 0.003, I2 =0%).

From the two groups above, it can be concluded that combination therapy is preferable to ABZ-only therapy for both three months and six months.

**Figure 7:**
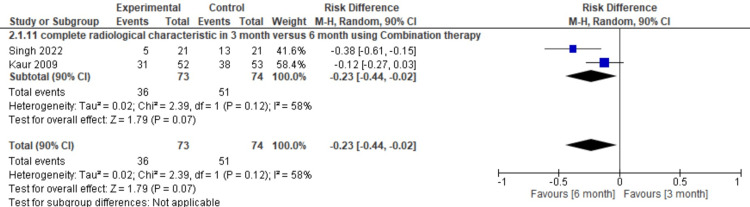
Meta-analysis for complete resolution in six months versus three months References: [13,14] Image Credit: Author Farida Ulfa

Furthermore, we wanted to see the effectiveness of ABZ and PZQ combination therapy in producing complete radiological characteristics using therapy for three months compared with six months (pooled RD = -0.23, 95% CI = -0.44--0.02, p = 0.12, I2 =58%). The results of this meta-analysis show that the combination therapy using ABZ and PZQ is better at eradicating neurocysticercosis lesions in the brain parenchyma compared to ABZ monotherapy. The I2 result of 58% was obtained, so this conclusion was not statistically meaningful; the sensitivity analysis step could not be carried out because it only had two papers (Figure 7).

**Figure 8:**
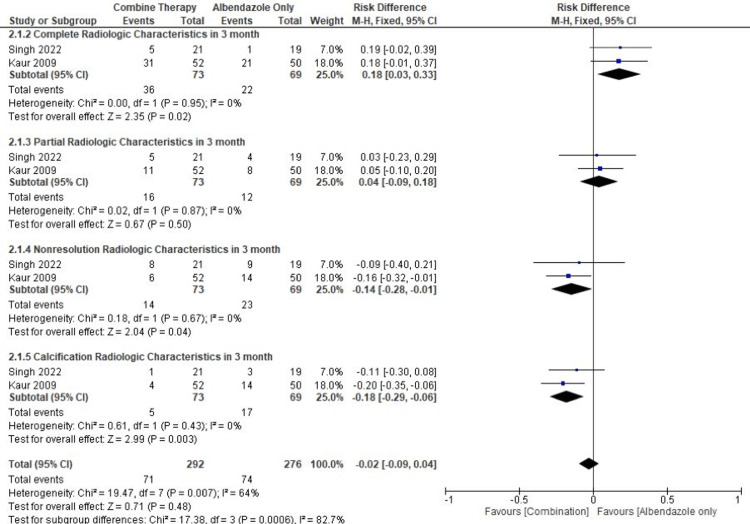
Meta-analysis based on the characteristics of lesion resolution after three months of therapy References: [13,14] Image Credit: Author Dian Andriani Ratna Dewi

In the NCC partial cure, there was no significant difference between the combination therapy group and the ABZ-alone therapy group (pooled RD = 0.04, 95% CI = -0.09-0.18, p = 0.50, I2 = 0%). Furthermore, from the results of the meta-analysis, the outcome of therapy that did not provide resolution of NCC lesions was significant in the combination group compared to the ABZ-alone therapy group (pooled RD = -0.14, 95% CI: -0.28--0.01, p = 0.04, I2 = 0%). However, the outcome of therapy that resulted in calcification of NCC lesions was significant in the combination group compared to the ABZ-alone therapy group (pooled RD = -0.18, 95% CI: -0.29--0.06, p = 0.0003, I2 = 0%) (Figure 8).

**Figure 9:**
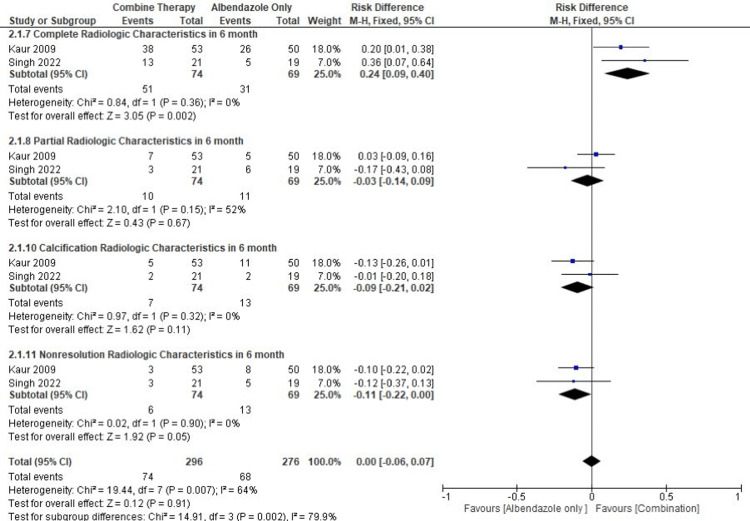
Meta-analysis based on the characteristics of lesion resolution after six months of therapy References: [13,14] Image Credit: Author Lila Irawati Tjahjo Widuri

From the results of the meta-analysis specifically for the treatment range for six months, it was found that there was a statistical significance for combination therapy being superior to cure NCC completely (pooled RD = 0.24, 95% CI = 0.09-0.40, p = 0.002, I2 =0%). Our meta-analysis showed non-significant results in intervention groups for the partial cure of NCC and non-significant results in therapies that caused calcification of NCC lesions and in therapies that did not result in the resolution of NCC lesions as depicted in radiology (Figure 9).

Strength and limitation

This is the first study to discuss the combination of ABZ and PZQ therapy versus ABZ monotherapy for lesion resolution in NCC in children. The shortcomings of this research are the limited number of studies that could be used, and the quality of the studies had some concerns overall.

## Conclusions

Comparing the efficacy of ABZ alone against ABZ and PZQ combination therapy for lesion clearance in pediatric NCC was the aim of this systematic review and meta-analysis. Complete lesion resolution outcomes were best achieved with combination therapy, according to the systematic review and meta-analysis, both three and six months after treatment. However, the result of calcification was also more significant in combination therapy, so clinical caution should be taken to prevent calcification during treatment. Future studies need to examine the effects of combination therapy versus monotherapy with high-quality randomized studies, more homogenous study designs, and a larger population.

## Appendices

Appendix 1: study selection keywords

PubMed

**Table 2:** Pubmed

No.	Search	Result
#1	((Neurocysticercosis [Title/Abstract]) AND (children[Title/Abstract])) Neurocysticercosis (MeSH Terms)	185
#2	((Praziquantel) AND (Albendazole)) AND (combination)	222
#3	#1 AND #2	4

Total: 4

Cochrane

**Table 3:** Cochrane

No.	Search	Result
#1	(Praziquantel) AND (Albendazole)	115
#2	“NEUROCYSTICERCOSIS”	124
#3	combination OR mix antiparasitic drug	190674
#4	Elderly OR Children OR Aged	781382
#5	MeSH descriptor: [Aged] in all MeSH products	255705
#6	#1 AND #2 AND #3 AND #4 AND #5	2

Total: 2

Google Scholar

((((Neurocysticercosis [Title/Abstract]) AND (children [Title/Abstract]))) Neurocysticercosis (MeSH Terms) AND ((Praziquantel) AND (Albendazole)) AND (combination))

Total: 7

Appendix 2: data extraction

**Table 4:** Data extraction

Author, year, country			Kaur et al., 2009, India [14]	Singh et al., 2022, India [13]
Title			Combination therapy with albendazole and praziquantel versus albendazole alone in children with seizures and single lesion neurocysticercosis: a randomized, placebo-controlled double-blind trial	Efficacy of combination therapy of albendazole and praziquantel vs albendazole monotherapy in children with persistent neurocysticercosis: a randomized controlled trial
Sample size, n (combined/albendazole only)			103 (53/50)	40 (21/19)
Age (Mean ± SD) or range of age	Combined		7.2 ± 0.76	5-14 years old
	Albendazole only		7.3 ± 0.84	5-14 years old
Adverse event			Three children in group A and two in group B developed headaches on days three to four of treatment lasting for one to two days.	No significant side effects
Design of the study			Randomized controlled trial (RCT)	RCT
Resolution of lesions in three months	Intervention (combination therapy)	Complete resolution / number of participants	31/52	7/21
		Partial resolution / number of participants	11/52	5/21
		Non Resolution resolution / number of participants	6/52	7/21
		Calcification resolution / number of participants	4/52	1/21
	Control (albendazole only therapy)	Complete resolution / number of participants	21/50	1/19
		Partial resolution / number of participants	8/50	4/19
		Non Resolution resolution / number of participants	14/50	9/19
		Calcification resolution / number of participants	7/50	3/19
Resolution of lesions in six months	Intervention (combination therapy)	Complete resolution / number of participants	38/53	13/21
		Partial resolution / number of participants	7/53	3/21
		Non Resolution resolution / number of participants	3/53	3/21
		Calcification resolution / number of participants	5/53	2/21
	Control (albendazole only therapy)	Complete resolution / number of participants	26/50	5/19
		Partial resolution / number of participants	5/50	6/19
		Non Resolution resolution / number of participants	8/50	5/19
		Calcification resolution / number of participants	11/50	2/19

## References

[1] Nash TE, Garcia HH (2011). Diagnosis and treatment of neurocysticercosis. Nat Rev Neurol.

[2] Dutta D, Kumar M, Ghosh S, Mukhopadhyay S, Chowdhury S (2013). Pituitary hormone deficiency due to racemose neurocysticercosis. Lancet Diabetes Endocrinol.

[3] Garcia HH, Nash TE, Del Brutto OH (2014). Clinical symptoms, diagnosis, and treatment of neurocysticercosis. Lancet Neurol.

[4] Aung AK, Spelman DW (2016). Taenia solium taeniasis and cysticercosis in Southeast Asia. Am J Trop Med Hyg.

[5] Gonzales I, Rivera JT, Garcia HH (2016). Pathogenesis of Taenia solium taeniasis and cysticercosis. Parasite Immunol.

[6] Garcia HH, Gonzalez AE, Gilman RH (2020). Taenia solium cysticercosis and its impact in neurological disease. Clin Microbiol Rev.

[7] Mahanty S, Orrego MA, Mayta H (2015). Post-treatment vascular leakage and inflammatory responses around brain cysts in porcine neurocysticercosis. PLoS Negl Trop Dis.

[8] El-Kady AM, Allemailem KS, Almatroudi A, Abler B, Elsayed M (2021). Psychiatric disorders of neurocysticercosis: narrative review. Neuropsychiatr Dis Treat.

[9] Monteiro L, Almeida-Pinto J, Stocker A, Sampaio-Silva M (1993). Active neurocysticercosis, parenchymal and extraparenchymal: a study of 38 patients. J Neurol.

[10] Hamamoto Filho PT, Rodríguez-Rivas R, Fleury A (2022). Neurocysticercosis: a review into treatment options, indications, and their efficacy. Res Rep Trop Med.

[11] Prodjinotho UF, Lema J, Lacorcia M (2020). Host immune responses during Taenia solium neurocysticercosis infection and treatment. PLoS Negl Trop Dis.

[12] Garcia HH (2008). Antiparasitic drugs in neurocysticercosis: albendazole or praziquantel?. Expert Rev Anti Infect Ther.

[13] Singh K, Saini AG, Khandelwal N, Singhi P (2022). Efficacy of combination therapy of albendazole and praziquantel vs albendazole monotherapy in children with persistent neurocysticercosis: a randomized controlled trial. J Child Neurol.

[14] Kaur S, Singhi P, Singhi S, Khandelwal N (2009). Combination therapy with albendazole and praziquantel versus albendazole alone in children with seizures and single lesion neurocysticercosis: a randomized, placebo-controlled double blind trial. Pediatr Infect Dis J.

[15] Morris DL (1987). Pre-operative albendazole therapy for hydatid cyst. Br J Surg.

[16] (2008). Small Animal Clinical Pharmacology. The Veterinary Journal.

[17] Venkatesan P (1998). Albendazole. J Antimicrob Chemother.

[18] Barrowman MM, Marriner SE, Bogan JA (1984). The binding and subsequent inhibition of tubulin polymerization in Ascaris suum (in vitro) by benzimidazole anthelmintics. Biochem Pharmacol.

[19] Cottingham K (2010). A new PTM for tubulin is found in a parasite. J Proteome Res.

[20] Bijman MN, van Nieuw Amerongen GP, Laurens N, van Hinsbergh VW, Boven E (2006). Microtubule-targeting agents inhibit angiogenesis at subtoxic concentrations, a process associated with inhibition of Rac1 and Cdc42 activity and changes in the endothelial cytoskeleton. Mol Cancer Ther.

[21] Akiyoshi DE, Weiss LM, Feng X, Williams BA, Keeling PJ, Zhang Q, Tzipori S (2007). Analysis of the beta-tubulin genes from Enterocytozoon bieneusi isolates from a human and rhesus macaque. J Eukaryot Microbiol.

[22] Aruleba RT, Adekiya TA, Oyinloye BE, Masamba P, Mbatha LS, Pretorius A, Kappo AP (2019). PZQ therapy: how close are we in the development of effective alternative anti-schistosomal drugs?. Infect Disord Drug Targets.

[23] Utzinger J, N'Goran EK, N'Dri A, Lengeler C, Tanner M (2000). Efficacy of praziquantel against Schistosoma mansoni with particular consideration for intensity of infection. Trop Med Int Health.

[24] Pax R, Bennett JL, Fetterer R (1978). A benzodiazepine derivative and praziquantel: effects on musculature of Schistosoma mansoni and Schistosoma japonicum. Naunyn Schmiedebergs Arch Pharmacol.

[25] Kohn AB, Anderson PA, Roberts-Misterly JM, Greenberg RM (2001). Schistosome calcium channel beta subunits: unusual modulatory effects and potential role in the action of the antischistosomal drug praziquantel. J Biol Chem.

[26] Doenhoff MJ, Cioli D, Utzinger J (2008). Praziquantel: mechanisms of action, resistance and new derivatives for schistosomiasis. Curr Opin Infect Dis.

[27] Doenhoff MJ, Sabah AA, Fletcher C, Webbe G, Bain J (1987). Evidence for an immune-dependent action of praziquantel on Schistosoma mansoni in mice. Trans R Soc Trop Med Hyg.

[28] Harnett W, Kusel JR (1986). Increased exposure of parasite antigens at the surface of adult male Schistosoma mansoni exposed to praziquantel in vitro. Parasitology.

[29] Brindley PJ, Strand M, Norden AP, Sher A (1989). Role of host antibody in the chemotherapeutic action of praziquantel against Schistosoma mansoni: identification of target antigens. Mol Biochem Parasitol.

[30] Matthaiou DK, Panos G, Adamidi ES, Falagas ME (2008). Albendazole versus praziquantel in the treatment of neurocysticercosis: a meta-analysis of comparative trials. PLoS Negl Trop Dis.

[31] White AC Jr, Coyle CM, Rajshekhar V (2018). Diagnosis and treatment of neurocysticercosis: 2017 clinical practice guidelines by the Infectious Diseases Society of America (IDSA) and the American Society of Tropical Medicine and Hygiene (ASTMH). Clin Infect Dis.

[32] Pretell EJ, García HH, Gilman RH, Saavedra H, Martinez M (2001). Failure of one-day praziquantel treatment in patients with multiple neurocysticercosis lesions. Clin Neurol Neurosurg.

[33] Escobedo F, Penagos P, Rodriguez J, Sotelo J (1987). Albendazole therapy for neurocysticercosis. Arch Intern Med.

[34] Garcia HH, Gilman RH, Horton J (1997). Albendazole therapy for neurocysticercosis: a prospective double-blind trial comparing 7 versus 14 days of treatment. Neurology.

[35] Hernandez LP, White Jr. AC (2017). Helminthic diseases: taeniasis and cysticercosis due to Taenia solium. International Encyclopedia of Public Health.

